# Robotic Assisted-Bronchoscopy With Cone-Beam CT ICG Dye Marking for Lung Nodule Localization: Experience Beyond USA

**DOI:** 10.3389/fsurg.2022.943531

**Published:** 2022-06-28

**Authors:** Joyce W. Y. Chan, Aliss T. C. Chang, Peter S. Y. Yu, Rainbow W. H. Lau, Calvin S. H. Ng

**Affiliations:** Division of Cardiothoracic Surgery, Department of Surgery, Prince of Wales Hospital, The Chinese University of Hong Kong, Hong Kong, China

**Keywords:** robotic bronchoscopy, ICG=indocyanine green, dye marking, hybrid operating room, fluorescence dye

## Abstract

Electromagnetic navigation bronchoscopy (ENB)-guided indocyanine green (ICG) fluorescence dye marking of subsolid, small and deep lung lesions facilitates subsequent minimally invasive lung resection surgeries. The novel robotic-assisted bronchoscopy (RAB) platform can improve the accuracy and yield of ENB biopsy, and the use of RAB has been extended to ICG dye marking. However, performing this procedure in the hybrid operating room guided by cone-beam CT (CBCT) with immediate proceed to lung surgery has not been well reported. We studied the safety, feasibility and clinical outcomes of 5 consecutive cases performed between December 2021 and March 2022. Navigation success was 100% while localization success using ICG was 80%. The benefits and pitfalls of robotic bronchoscopy procedures, and challenges of combining with hybrid operating room CBCT were discussed in detail. In conclusion, robotic-assisted bronchoscopy is a promising and useful tool for ICG fluorescence dye-marking, providing accurate navigation, superior maneuverability and improved ergonomics compared to conventional bronchoscopy-guided ENB procedures. Learning curve is reasonable, but meticulous system set up to incorporate the robotic system into existing CBCT platform may be required to ensure a smooth procedure.

## Introduction

With growing evidence of efficacy of lung cancer screening (1) and increasing availability of computer tomography (CT) scans, rising numbers of small, deep and subsolid lung nodules are being incidentally discovered, many of which harbour early malignant changes. Electromagnetic navigation bronchoscopy (ENB) has been utilized for intraoperative fluorescent dye-marking of these lesions followed by subsequent surgery (2–4). Robotic Assisted-bronchoscopy (RAB) is a novel technology serving the same purpose, with the possibility of improved accuracy, efficiency and ergonomics (5, 6). We present below the first outside-of-USA experience of using robotic bronchoscopy (Monarch™ platform by Auris Health Inc.) for indocyanine green (ICG) dye marking of lung nodules. Furthermore, we discuss challenges encountered in our patient population and when used with cone-beam CT (CBCT) in our hybrid operating suite followed by immediate surgery.

## Rationale for Robotic Bronchoscopy Fluorescent Dye Marking

Robotic assisted-bronchoscopy (RAB) was initially developed to improve the accuracy and yield of lung nodule biopsy. Compared to conventional bronchoscopy, or other forms of guided bronchoscopy, including virtual bronchoscopy (VB), radial endobronchial ultrasound (r-EBUS) and electromagnetic navigation bronchoscopy (ENB), robotic bronchoscopy has the advantage of direct visualization of airways and deeper navigation depth, up to the 9th airway generations (7). The Monarch™ platform used in our unit includes an outer sheath and an inner bronchoscope with a 4-way steering control, allowing superior maneuverability than conventional bronchoscope. Structural support was provided by the RAB system while the inner scope was advanced, reducing the likelihood of scope prolapse in proximal airways. In cadaveric and early human trials, RAB biopsy has been shown to provide excellent navigation with success up to 88%–97%, and diagnostic yield of 77%–97% (8–10), especially when combined with r-EBUS. Diagnostic yield in lesions without bronchus sign was reported to be 54%, which was also higher than the previously reported yield of 31%–44% using non-robotic systems (11). The improved accuracy of RAB may be attributable to the fact that it provides the ability to maneuver and control the distal end of the scope, and the ability to aim instruments with better precision through controlling multiple active articulation points within the scope. We extend the use of RAB to dye marking of non-palpable lung lesions for subsequent lung resection. Its ability to inject dye closer to the target lesion could also aid intra-operative visualization of dye, better margin determination, and more lung preservation.

Localization of small, deep, subsolid or ground-glass lesions during video-assisted thoracoscopic surgery (VATS) is challenging, as they do not alter the surface of visceral pleura and palpation is limited by the small wound. Numerous pre-operative or intra-operative localization techniques have been described, including hookwires, microcoils, coloured dye, iodinated contrast, radioactive markers and fiducial markers. In recent years, ICG fluorescence has been an important adjunct as a marker during VATS, as it is capable of deep tissue penetration (12) and can be distinguished regardless of lung anthracosis. Intraoperatively, the ICG dye can be visualized using near-infrared thoracoscope, and the dye is detectable at up to 2.4 cm beneath the surface of visceral pleura (13). Dye injection can be performed either percutaneously or transbronchial, with the latter sparing pleural puncture and therefore reducing the risk of pneumothorax. Small pneumothoraxes may not require chest drain insertion, but they prevent repeated percutaneous marking procedures, thus CT-guided percutaneous approach is less than ideal for marking multiple lung lesions. On the contrary, the transbronchial approach allows the marking of multiple lesions without injuring the pleura and can access regions that are otherwise difficult or dangerous to reach *via* the percutaneous approach, for example, regions close to the mediastinum or shielded by bony structures. In order to yield the benefits of transbronchial marking, accuracy in reaching lung lesions is of utmost importance, which can be ensured by combining it with robotic bronchoscopy under ENB guidance.

## Our Institute's Initial Experience in Robotic Bronchoscopy Hybrid Operating Room CBCT Image-Guided ICG Dye Marking of Lung Nodules

**Figure 1:**
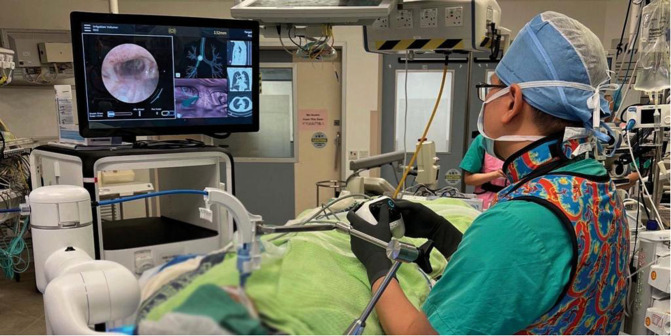
The operator commands the outer sheath and inner bronchoscope *via* a controller. On the monitor, the left side panel shows the real-time bronchoscopic images, while the right side panels show virtual bronchoscopic and various choices of other views to aid navigation and localization.

**Figure 2:**
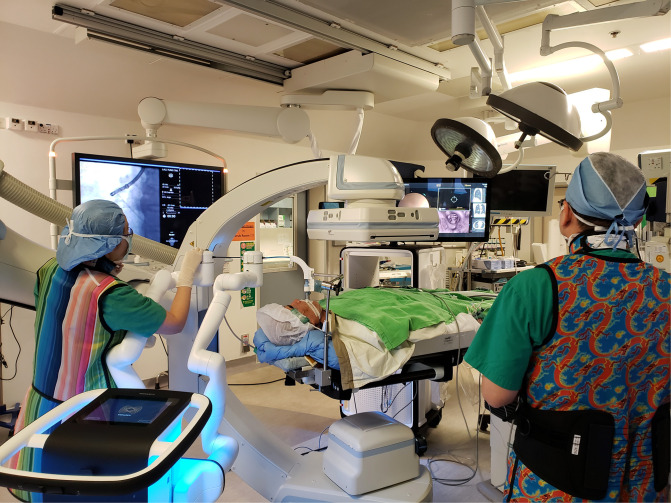
Patient lies supine, intubated by single-lumen endotracheal tube under general anaesthesia. The robotic bronchoscope enters the endotracheal tube *via* the head-side. Robotic bronchoscopy guided by CBCT in our hybrid operating room is usually performed by two operators. One operator (right) performs fine-tuning of the direction of bronchoscope using the controller console, another operator (left) controls the advancement of instruments and commands fluoroscopic screening using different angles of C-arm. The lesion is usually marked on the fluoroscopy screen using i-guide® CBCT platform so that the relationship between instruments and lesion can be seen on live fluoroscopy. In this figure, the robotic arm can be seen in very close proximity with the C-arm. Both the robotic platform monitor and the fluoroscopy screen should be visible to both operators, and communication is important for coordination.

**Figure 3:**
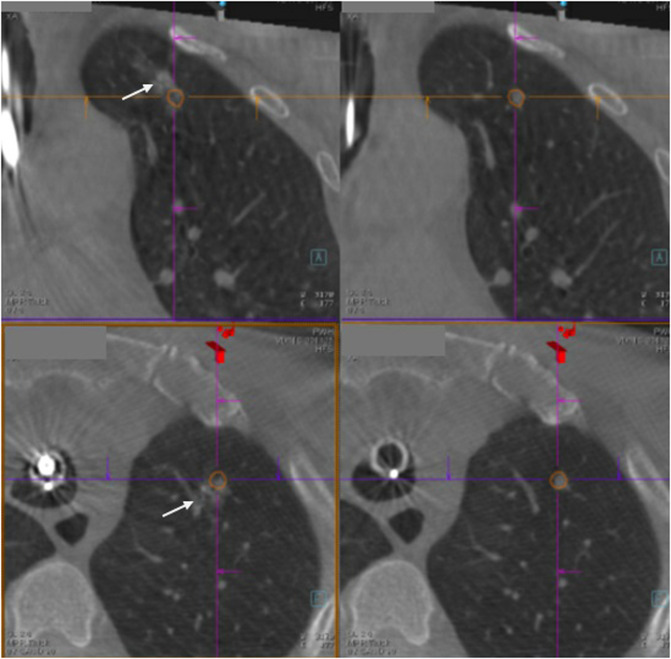
The right-sided panels show the lung lesion marked by orange tracings, while the left-sided panels show triple dye (arrow) (which contains iodinated contrast, indocyanine green and methylene blue) injected close to the lung lesion and within 2 cm from the pleural surface.

**Figure 4:**
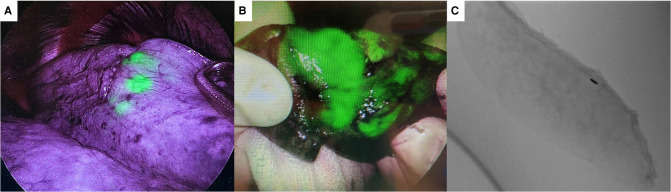
(**A**) shows indocyanine green (ICG) dye visible through the pleural surface during video-assisted thoracoscopic surgery using ICG fluorescence thoracoscope. (**B**) shows ICG dye on a wedged specimen. (**C**) shows fiducial marker in specimen side ensuring adequate deep margin achieved.

Between December 2021 and March 2022, we performed robotic bronchoscopy ICG dye-marking for 5 lung nodules using CBCT image guidance in our hybrid operating room. All cases were performed using the Monarch™ robotic platform under general anaesthesia (Figures 1 and 2), followed immediately by same session VATS in the hybrid operating room for wedge resection with or without proceeding to lobectomy after frozen section. Navigation to the lesion by robotic bronchoscopy was aided by fluoroscopic overlay and CBCT. “Triple dye” was injected into or next to lesions, containing equal volumes of indocyanine green (ICG), methylene blue and iodinated contrast (4). The location of the dye was confirmed during injection using real-time fluoroscopy, while the position of iodinated contrast in relation to the lesion was visualized with CBCT scan (Figure 3). Intraoperatively, the dye was then identified during VATS using both white light and near-infrared light (ICG thoracoscope) (Figure 4). In 4 out of 5 patients, an additional fiducial marker (Superlock™ Nitinol Coil, Medtronic) was placed 2 cm proximal to lesions *via* the same bronchoscopic delivery system to mark the deep margin (14). Fluoroscopy was used during VATS to ensure the inclusion of fiducial markers on the specimen side before firing of staples (Figure 4C).

The baseline characteristics and clinical outcomes of our case series can be seen in Table 1. The mean size of nodules was 8.3 mm, ranging from 3.5–14 mm. Four out of 5 nodules were of Suzuki class I or II ground glass lesions without solid components, at a mean of 9.3 mm from the pleura. The remaining lung nodule was solid but was only 4 mm in diameter and 15.6 mm from the pleural surface. In three out of 5 cases, the dye-marked lesion was the secondary lesion, as surgery was performed for lobectomy of the primary larger lesion in a different lobe on the same side. Using robotic bronchoscopy, the needle was navigated towards the lesion and was able to reach within 1 cm of the lesion in all cases. Dye injection location was confirmed on CBCT to be satisfactory in all cases. However, in 1 case the fiducial marker was dislodged prematurely during manipulation of the delivery system, such that the marker was placed approximately 1 cm more proximal than desired. During VATS, eventual localization was successful in all cases, although neither ICG nor methylene blue was initially visible in the last case. This was attributable to a relatively small volume of dye being injected (0.2 mL, in comparison to the usual 0.5 mL), and a relatively deeper location of dye (2.3 cm below the pleural surface). The nodule was localized eventually by identifying the fiducial marker under fluoroscopy. Mean operative time (including both dye marking and VATS) was 262 min (range 204–332 min), which was slightly longer than the usual dye marking procedures using ENB alone, reflecting an initial learning curve. Patients were discharged between the 2nd and 5th post-operative day. No significant complications occurred in relation to dye marking. The above data showed that robotic bronchoscopy ICG dye marking with image guidance by CBCT in the hybrid operating room setting followed immediately by VATS is feasible, safe and has a high rate of localization success.

**Table 1:** Baseline characteristics and clinical outcomes of robotic bronchoscopy ICG dye marking cases.

Case	Location	Diameter (mm)	Suzuki class	Distance from pleura (mm)	Dye marking	Fiducial Marker	Procedure	Operation duration (minutes)	Needle tip to lesion distance (mm)	Navigation success	Localization success
1	RUL	3.5	2	13	+	−	RUL + RLL wedge	204	8	+	+
2	LUL	4	6	15.6	+	+	LUL + LLL wedge	210	4	+	+
3	RLL	10	2	13	+	+	RLL wedge then lobectomy	313	5	+	+
4	LLL	14	2	5	+	+	LLL apical segmentectomy	253	10	+	+
5	LLL	10	1	0	+	+	LUL lobectomy + LLL wedge	332	8	+	+/−^a^

*RUL, right upper lobe; LUL, left upper lobe; RLL, right lower lobe; LLL, left lower lobe.*

*^a^* *Intraoperatively, neither ICG nor methylene blue dye were visualized, but eventual localization was successful using fiducial marker position under fluoroscopy screening.*

## Discussion

Our institute has been performing ENB-related procedures including biopsy (15–17), dye marking (2–4) and microwave ablation (18) using Artis Zeego with PURE® CBCT platform (Siemens Healthineers, Germany) since 2015. We launched the robotic bronchoscopy program since December 2021 and became the first centre outside of the United States to use the Monarch™ robotic platform, in the hope of deriving the benefits of improved accuracy and ergonomics during the procedure. Unlike other RAB centres, we have performed all our cases in the hybrid operating room aided by overlay fluoroscopy and CBCT. Over 30 cases of RAB-guided biopsy and/or dye marking has been performed, and we find that the system is a great aid to our procedures although there was an initial learning curve and meticulous system setup was crucial to ensure smooth procedure.

The robotic platform improves the ability of the surgeon to confidently localize small lung nodules, as the bronchoscope can be advanced into more peripheral bronchi, up to 4.2 cm further (7), than the conventional bronchoscope. The unique sheath telescoping design provides excellent stability and control. The sheath can be articulated up to 130 degrees in any direction to create a stable base, while upon advancement the inner bronchoscope can flex an additional 180 degrees in any direction, allowing tricky navigation through small and tortuous bronchi. Each component of the bronchoscope can be independently articulated, advanced, retracted and positionally locked. The system also provides the ability to control and release tension in the system. More support is provided by the robotic system, as the relatively rigid outer sheath provides stability of the scope relative to airways when the inner scope is advanced. This is in contrast to the conventional ENB procedures where the tip of the bronchoscope is the only controllable part, and further fine-tuning of direction is provided by the pre-formed curvature of the tip of the extended working channel, the direction and curvature of which cannot be independently controlled. The tension of the body of conventional bronchoscope is generally not under the operator's control but rather is determined by the pathway and angle taken by the bronchoscope.

Superior vision from RAB is enabled by an integrated camera and irrigation and suction channel built into the bronchoscope. Direct visualization of airways enables the operator to perform fine adjustment of the direction of the tip such that instruments can be directed into desired branches under direct vision without the need of blind trial-and-error which is at times required in conventional ENB procedures. In addition, the advancement and flexing of the bronchoscope is done *via* a controller console, which we find quite intuitive. Minimal training is required before being able to master the control of bronchoscope. Previously, without robotic control, the operator often needs to raise the left arm high above the shoulder and twist in awkward angles in order to control the direction of conventional bronchoscopes, and it is not uncommon for operators to suffer from muscle fatigue, shoulder or thumb pain after prolonged procedures.

In RAB procedures, lesions were reached by the guidance of electromagnetic navigation, which matches the virtual bronchoscopy images generated by the pre-operative CT scan with the patient's actual bronchial anatomy during the procedure.

In the peripheral navigation view, the target lesion is represented by a yellow ball, and in theory, the instrument should be pointing directly at the lesion if the yellow ball is shown in the centre of the screen. However, from our experience, there is sometimes some degree of disparity, often within 1–2 cm, between the navigation platform and reality. This is the CT-to-body divergence which may be due to atelectasis and breathing movements of the patient. Therefore, it is our common practice to utilize CBCT during the procedure to confirm accurate needle placement (16) or to aid fine adjustment of instrument tip after navigation. We also use fluoroscopy and overlay function at different angles to aid final adjustment of instrument direction, as electromagnetic navigation accuracy may be affected when CBCT C-arm is close by.

**Figure 5:**
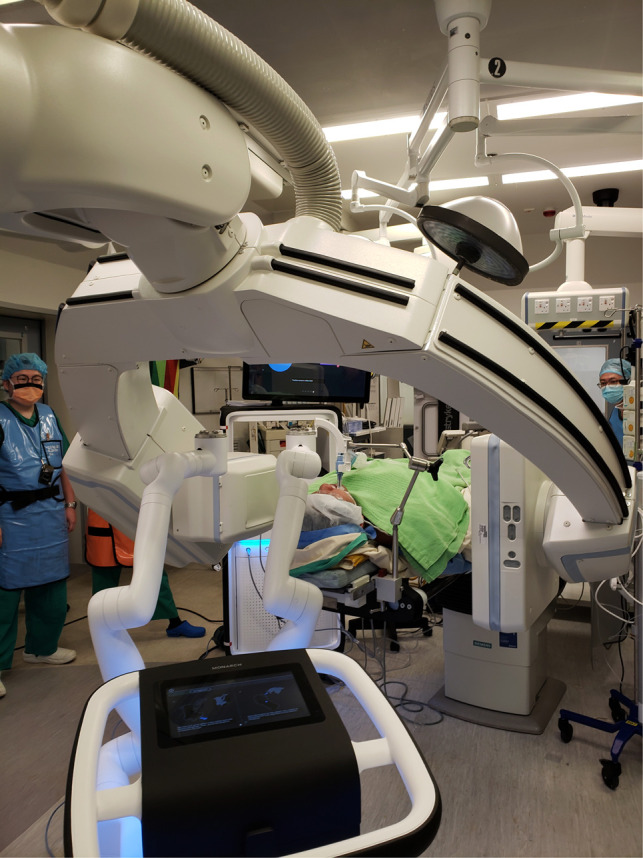
The left robotic arm can be seen in very close proximity to the cone-beam CT (CBCT) C-arm during CBCT spin. The metallic holder of the endotracheal tube is also close to the other side of the CBCT C-arm. Care must be taken to avoid collisions in these two regions.

**Figure 6:**
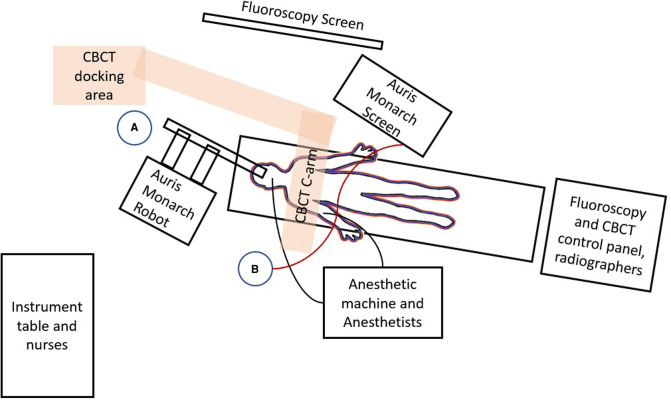
Floor plan of the hybrid operating room setup for robotic ENB procedures in our institution is shown. The patient lies on a radiolucent operating table and is intubated and connected to the anesthetic machine on his right side *via* numerous monitoring lines. The Auris Monarch Robot is parked at the head-side of the table with robotic bronchoscope entering the airways through the endotracheal tube. The fluoroscopy screen, and the Auris Monarch Screen showing virtual bronchoscopy and navigation pathways, is parked on the patient's left. The CBCT (orange rectangle overlying the patient's thorax) rotates around the patient, while the CBCT docking area is fixed to the ground. Operator A stands close to the Robot in order to control instruments within the robotic bronchoscope, for example advancing needle or injecting dye. Operation B stands at the patient's right side and controls the advancement and direction of the robotic bronchoscope *via* a wired steering control (red line) which is connected to the Auris Monarch Machine/screen. Both operators should see both the fluoroscopy and Auris Monarch screen and remain in close communication with each other.

The CBCT system in our hybrid operating room was Artis Zeego (Siemens Healthineers), which we have worked with for more than 7 years. However, in the initial few cases of RAB, we encountered significant difficulties in incorporating both the CBCT C-arm and the robotic machine in a position where collision can be avoided during CBCT spin. The robotic arms, which needs to be directed at 60 degrees to the plane of the endotracheal tube, at its fullest retraction is often in way of the CBCT spin (Figure 5). This is especially aggravated by the fact that airways are shorter in Asians than Caucasians, such that a longer length of robotic bronchoscope and arms are exposed externally. We developed several solutions, which include floor markings to standardize the best parking location of the RAB machine, removing the suction/irrigation cap to gain an additional 2 cm clearance space, and advancing the outer sheath of bronchoscope further into the airways such that the robotic arms are less retracted externally. A floor plan of our hybrid operating room setting is detailed in Figure 6. Indeed, the setup would be unique in every hybrid operating room, as different operating tables, robotic platforms and CBCT platforms are being used. Ideally a mobile low-profile C-arm with CT function, for example, the Cios Spin® (Siemens Healthineers) system should avoid most of the collisions (17).

Future designs or modifications of robotic bronchoscope may take into consideration of the shorter airways in our population, such that shorter robotic bronchoscopes would suffice. Slimmer bronchoscopes will further enhance our ability to reach more distal lesions, although this may limit the choice of instruments applicable and the suction clearance ability of the bronchoscope. Robotic arms may be designed such that they can be inserted from the head-side at midline so that the external parts would not interfere with the CBCT spin path.

Placement of SuperLock™ fiducial markers using the robotic platform also requires extreme caution and understanding of the fact that the distal part of the delivery catheter is unsupported, making premature or unintended deployment of fiducial markers possible. In conventional ENB setting, the extended working channel (EWC) is advanced to the desired location of marker deployment, and the fiducial marker catheter is placed within and supported by the relatively rigid EWC (14, 19). Marker deployment usually involves minimal manipulation of the delivery catheter tip. However, since the commercially available EWC is too thick to enter the robotic bronchoscope working channel, and the tip of bronchoscope is usually not distal enough at the desired deployment site, the fiducial marker delivery catheter is inserted through the bronchoscope and further manipulation of the tip direction is required to ensure proper marker deployment. The flexible and unsupported catheter tip flips around easily in the distal airways and the centrifugal force can cause premature deployment of the fiducial marker out of the catheter without any pushing force.

This procedure is limited by the availability of robotic bronchoscopy platform, and the need for image accuracy provided by fluoroscopy and CBCT in addition to robotic ENB navigation. Hybrid operating rooms may not be available in most hospitals, and while fluoroscopy and mobile CBCT machines may help, these could be limited by the lack of physical space in an ordinary operating theatre. The hybrid operating room also requires a high initial setup cost and subsequent maintenance cost, in addition to the cost of the robotic platform itself.

## Conclusion

Robotic-assisted bronchoscopy is a promising and useful armamentarium for ICG fluorescence dye-marking of lung nodules prior to video-assisted thoracoscopic surgeries. It provides accurate navigation, superior maneuverability and improved ergonomics compared to conventional bronchoscopy-guided ENB procedures. Learning curve is the reasonable but meticulous system set up to incorporate the robotic system into the existing CBCT platform may be required to enable smooth procedure.

## Data Availability

The raw data supporting the conclusions of this article will be made available by the authors, without undue reservation.
